# Assessing basic life support awareness, and knowledge among university undergraduates: a cross-sectional study

**DOI:** 10.1097/MS9.0000000000003076

**Published:** 2025-02-28

**Authors:** Mohamed Baklola, Mohammed Elnemr, Mohamed Ghazy, Yusof Omar, Nour Edin Darwish, Baher Ahmed, Eman Alkalla, Reem Reda, Hanaa Elmimy, Teeb Mohamed, Aya Sherif, Naji Al-Bawah, Dina Gamal Shaheen

**Affiliations:** a Faculty of Medicine, Mansoura University, Mansoura, Egypt; b Faculty of Medicine, Sana’a University, Ssana’a, Yemen; c Public Health and Community Medicine Department, Faculty of Medicine, Mansoura University, Mansoura, Egypt

**Keywords:** basic life support, BLS knowledge, CPR training, gender disparities, university students

## Abstract

**Background:**

Basic life support (BLS) is a critical skill for saving lives during emergencies. This study aims to assess BLS awareness and knowledge among undergraduates at University, examining the impact of gender, faculty, and prior correct cardiopulmonary resuscitation (CPR) training on students’ confidence and competence.

**Methods:**

A descriptive, cross-sectional study was conducted from August to December 2023 across six faculties: medicine, nursing, engineering, sciences, commerce, and law. Using convenience sampling, a total of 1165 students participated by completing a self-administered, bilingual questionnaire. Descriptive statistics, Pearson correlation tests, t-tests, ANOVA, and multivariate logistic regression analyses were performed using SPSS software version 26, with visual representations created using R software.

**Results:**

The majority of participants were female (64.8%), with a significant portion (70%) believing that CPR training should be mandatory. Only 29.6% had received CPR training. Females scored higher on BLS knowledge than males (4.9 vs. 4.5, *P* < 0.05). Students from medical faculties had greater BLS knowledge compared to their non-medical peers (6.3 vs. 3.5, *P* < 0.05). Trained students were more confident in performing CPR (35.1% vs. 10.2% among untrained, *P* < 0.05). Multivariate logistic regression identified prior CPR training and medical faculty enrollment as significant predictors of higher BLS knowledge and confidence.

**Conclusion:**

Significant gaps in BLS knowledge and training exist among University undergraduates, with disparities based on gender, faculty, and prior training. The findings underscore the need for mandatory CPR training across all faculties to enhance student preparedness and competence.

## Introduction

**Highlights**

- Evaluated BLS knowledge and confidence among Mansoura University undergraduates.
- Females and medical faculty students showed higher BLS knowledge scores.
- Trained students exhibited significantly higher BLS confidence and knowledge.
- 71.2% supported mandatory CPR training, though only 29.6% had prior training.
- Prior CPR training and medical faculty enrollment predicted better BLS knowledge.

Basic life support (BLS) is a set of emergency measures, generally done by any bystander, with the potential to save victims of life-threatening illnesses and injuries until medical intervention can be started.^[1]^. BLS involves the early detection of cardiac arrest, activation of emergency response systems, correct cardiopulmonary resuscitation (CPR), and rapid defibrillation to restore normal blood flow to essential organs, particularly the brain^[2]^. Timely BLS reduces mortality and morbidity in various medical emergencies such as out-of-hospital cardiac arrests (OHCAs) and severe road traffic accidents, both of which are leading causes of death in Egypt^[3-5]^.

OHCAs are predominantly caused by ischemic heart diseases^[6]^, which are the number one cause of death in Egypt^[7]^, and account for 20.3% of total deaths in the East Mediterranean Region^[8]^. Prompt BLS measures and automated external defibrillator application can double the survival rate of OHCA patients, as ventricular fibrillation is the primary cause of sudden cardiac death in nearly 60% of cases^[9]^.

Road traffic accidents are a major source of hospitalization and death in Egypt, accounting for 34% of non-fatal injuries and 63% of injury-related deaths in the country^[5]^. Nearly 12 000 Egyptians are lost every year because of road traffic accidents^[10]^, resulting in serious human and financial losses^[11]^. Studies show that BLS training effectively reduces mortality in victims of road traffic accidents^[12,13]^, especially in low-resource countries such as Egypt.

Given the role of BLS in significantly reducing mortality in OHCA patients and victims of road traffic accidents, the lack of BLS knowledge among both the general population and medical practitioners is a significant obstacle to improving survival rates in the country. The American Heart Association and the European Resuscitation Council both recommend that BLS training be made mandatory for all citizens to increase the likelihood of bystander CPR in cases of cardiac arrest^[14]^. This is further supported by studies showing that people who have undergone CPR training are more likely to perform bystander CPR^[15]^, and that the integration of CPR training into the curriculum is an effective measure in increasing knowledge of and comfort level with CPR^[16]^.

Despite the great importance placed on BLS education, the high prevalence of cardiovascular diseases, and the incidences of cardiac arrest in the Arab world, knowledge of BLS among the general population of Arab countries remains insufficient and moderate at best^[17]^. A recent multinational study in nine Arab countries^[17]^ showed that despite 30% of the population facing a person in need of BLS, only 47.3% of the study participants had ever heard of BLS, and only 23.8% of the participants had received BLS training. In Egypt, studies suggest that BLS knowledge among medical students, the country’s future healthcare providers, is inadequate^[18,19]^.

In a developing country such as Egypt, where cardiac diseases are the leading cause of death and accessibility of emergency medical services in cases of road traffic accidents is limited, research studying the prevalence of, as well as factors associated with, BLS knowledge among the general population can have a significant impact on reducing mortality and morbidity rates in the country.

The current study aimed to assess the level of BLS knowledge, awareness, and attitudes among undergraduate university students. The findings of this study can help identify gaps in BLS knowledge and suggest ways to improve BLS education among medical and nonmedical students. The results of this study can also be used to design effective BLS training programs that can be implemented in universities and other educational institutions, thereby contributing to the overall improvement of emergency medical care in the country and decreasing mortality due to cardiac diseases and OHCAs.

## Methods

### Study design and study period

A descriptive, cross-sectional study with an analytic component was conducted from August to December 2023 at our university in six faculties: medicine, nursing, engineering, sciences, commerce, and law. The study has been reported in line with the STROCSS (Strengthening the Reporting of Cohort, Cross-Sectional, and Case-Control Studies in Surgery) criteria to ensure transparency and consistency in reporting^[20]^.

### Sample size

The study’s sample size was determined using Medcalc 15.8, and we assumed a population proportion of 50%, a margin of error of 5%, and a confidence interval of 95%. The calculator indicated that we would need at least 385 participants. Considering the design effect of the proposed stratified cluster sampling method, this sample size was then multiplied by 3, resulting in a final required sample size of 1155 participants. 1165 participants completed the questionnaire.

### Sampling and data collection approach

The study employed a convenience sampling approach, which, while practical for reaching a large and diverse group of participants, may limit the generalizability of the findings to the broader population. The required sample size for each selected faculty was determined using a proportionate allocation technique to ensure representation across different academic disciplines. Data collection commenced on 1 August 2023, and continued until the sample was complete, utilizing Google Forms for questionnaire administration. The survey was distributed to all students through official groups on various social media platforms, including the Telegram app, allowing participants to respond anonymously and at their convenience. Additionally, study members took on the responsibility of data collection at each faculty, posting the questionnaire in official groups across all academic years, ensuring coverage, and reaching all students without imposing any obligation to participate. Participants were free to respond at their own pace and maintain anonymity throughout the process.

### Study tools

A self-administered, bilingual questionnaire in Arabic and English was utilized to collect data for the study. The questionnaire was adapted from a previously conducted study among university students in Saudi Arabia^[21]^, and consisted of 13 different items based on the American Heart Association’s BLS manual. The survey was divided into three sections, with the first section focusing on demographic details of the participants, e.g., sex, age, faculty, marital status, and academic year. The second section assessed CPR awareness and attitudes, self-confidence, and self-perception of sufficiency, while the third section assessed BLS knowledge and knowledge retention. Given the high incidence of road traffic accidents and injuries in Egypt, some questions in the survey pertained to trauma management cases. Each question in the survey was followed by four-choice options, with one answer being correct or best. The responses to individual items were summed to obtain a total score.

We conducted a pilot study on a sample of 39 students. Additionally, four experts with expertise in public health and emergency services evaluated the survey for clarity and reliability. The pilot study’s internal consistency was assessed using Cronbach’s α reliability coefficient, which indicated moderate to good reliability (Cronbach’s α = 0.68).

### Statistical analysis

Descriptive statistics were used to summarize the sociodemographic characteristics of the participants. Mean and standard deviation (SD) were calculated for continuous variables, while frequencies and percentages were used for categorical variables. BLS knowledge scores were calculated according to the survey responses. For inferential statistics, Pearson correlation tests were conducted to examine the relationships between sociodemographic variables, BLS knowledge, and confidence in performing CPR. Independent sample t-tests and ANOVA were used to compare mean BLS knowledge scores across different groups based on gender, faculty, and prior CPR training. Additionally, multivariate logistic regression analyses were performed to identify predictors of CPR performance and confidence levels among participants. R software was utilized to create visual representations of the data, including bar charts. All statistical analyses were conducted using SPSS software version 26, with a significance level set at *P* <0.05.

## Results

### Demographics and CPR training

Table 1 displays the demographics of the participants. 1165 individuals completed the questionnaire, comprising 410 males (35.2%) and 755 females (64.8%). Approximately two-thirds of the participants, 743 individuals (63.8%), were aged 20 or younger. Most participants were single (90.6%). within the participants, the largest subgroup consisted of third-year students (38.4%). Notably, a substantial proportion (70.4%) indicated that they had not received CPR training, while 29.6% reported having undergone such training. The vast majority (91.1%) had not encountered a situation necessitating CPR. Interestingly, most respondents (71.2%) believed that BLS training should be mandatory.

**Table 1:** Demographic characteristics, awareness, and attitudes toward CPR training and experience among undergraduate students (n = 1165)

Variables	Categories	Frequency n (%)
Age	Below or equal to 20	743 (63.8)
	Above 20	422 (36.2)
Sex	Female	755 (64.8)
	Male	410 (35.2)
Faculty	Medicine	309 (26.5)
	Nursing	226 (19.4)
	Engineering	160 (13.7)
	Sciences	168 (14.4)
	Commerce	146 (12.5)
	Law	156 (13.4)
Marital status	Single	1055 (90.6)
	Engaged	69 (5.9)
	Married	41 (3.5)
Academic year	First year	147 (12.6)
	Second year	277 (23.8)
	Third year	447 (38.4)
	Fourth year	121 (10.4)
	Fifth year	103 (8.8)
	Sixth year	11 (0.9)
	Intern	59 (5.1)
Have you ever received a course or training on CPR?	No	820 (70.4)
	Yes	345 (29.6)
If you have, when was it?	Before 2010	3 (0.9)
	2010–2015	40 (11.6)
	2016–2020	302 (87.5)
If you have, where did you receive it?	University-based Training	183 (53)
	External specialized organization training	87 (25.2)
	Others	75 (21.8)
Do you intend to attend another one in the future?	No	217 (25.1)
	Yes	646 (74.9)
Have you ever been in a situation that required you to do CPR?	No	1061 (91.1)
	Yes	104 (8.9)
If yes, did you perform CPR?	No	60 (58.3)
	Yes	43 (41.7)
Do you think a basic life support (BLS) course should be mandatory?	Yes	744 (71.2)
	No	45 (4.3)
	Maybe	256 (24.5)

### Basic life support (BLS) knowledge

Table 2 demonstrates significant variations in BLS knowledge scores among participants based on several factors. Females exhibited a notably higher mean BLS score of 4.9 (SD 2.9) compared to males, who averaged 4.5 (SD 3.1) (*P* < 0.05). Moreover, participants enrolled in medical faculties demonstrated a substantially greater mean score of 6.3 (SD 2.9) in contrast to their counterparts in non-medical faculties, who scored 3.5 (SD 2.4) (*P* < 0.05). Academic progression also influenced BLS knowledge, with third-year students achieving the highest mean score of 5.1 (SD 2.9) across all years (*P* < 0.05). Notably, individuals who received CPR training showed a significantly higher mean BLS score of 7.5 (SD 2.3) compared to those without training, who scored 3.6 (SD 2.4) (*P* < 0.05).

**Table 2:** Sociodemographic characteristics and mean of the correct responses attempted by the students of various faculties

Variables		BLS total score Mean (SD)	*P* value
Age	Below or equal to 20	4.7 (2.9)	0.1
	Above 20	5 (3.1)	
Sex	Female	4.9 (2.9)	<0.05
	Male	4.5 (3.1)	
Faculty	Medical	6.3 (2.9)	<0.05
	Non-medical	3.5 (2.4)	
Marital status	Single	4.8 (3)	0.06
	Engaged	4.9 (3.2)	
	Married	4.8 (2.9)	
Academic year	First year	3.6 (2.6)	<0.05
	Second year	4.8 (3)	
	Third year	5.1 (2.9)	
	Fourth year	3.9 (2.8)	
	Fifth year	5.1 (3.3)	
	Sixth year	6.2 (3.2)	
	Intern	6.2 (3.3)	
Have you ever received a course or training on CPR?	No	3.6 (2.4)	<0.05
	Yes	7.5 (2.3)	
Have you ever been in a situation that required you to do CPR?	No	4.7 (3)	<0.05
	Yes	5.6 (2.9)	

### Comparison of BLS knowledge between medical and non-medical participants

Table 3 presents a comparison of correct response rates among participants with medical and non-medical backgrounds across various BLS knowledge items. Medical participants significantly outperformed their non-medical counterparts in understanding how to respond to an unresponsive patient (90.1% vs. 66%, *P* < 0.05) and the importance of not immediately transferring accident victims to hospitals (44.7% vs. 23.5%, *P* < 0.05). They also exhibited greater knowledge in airway management for injured or burned patients, chest compressions during resuscitation, and management of choking incidents (all *P* < 0.05). Overall, medical participants achieved a higher mean total score of 6.3 (SD 2.9) compared to 3.5 (SD 2.4) among non-medical participants (*P* < 0.05).

**Table 3:** Comparisons between medical and non-medical students regarding items and total score of BLS knowledge

Items	Medical correct responses number (%)	Non-medical correct responses number (%)	*P* value
What is the ambulance number?	383 (71.6)	432 (68.6)	0.2
What will you do if you find a patient who is unresponsive?	482 (90.1)	416 (66)	<0.05
When you see a victim of an accident, you should quickly transfer them to a hospital without waiting for help.	239 (44.7)	148 (23.5)	<0.05
How is the airway opened in injured or burnt patients?	151 (28.2)	44 (7)	<0.05
If a person is unconscious, has a weak pulse, and is not breathing, what should we do?	118 (22.1)	107 (17)	<0.05
What should be done if the person didn’t know mouth-to-mouth ventilation or there is any limitation to perform it?	186 (34.8)	213 (33.8)	0.73
If we need to do chest compressions for resuscitating, what will be the location for chest compressions?	331 (61.9)	158 (25.1)	<0.05
While doing resuscitation, what will be the ratio of chest compression and ventilation in adults?	330 (61.7)	71 (11.3)	<0.05
What is the depth of chest compressions in resuscitation in adults?	242 (45.2)	82 (13)	<0.05
Which of the following is the most important intervention in resuscitation?	137 (25.6)	108 (17.1)	<0.05
How shall we manage severe and complete choking in adults?	240 (44.9)	121 (19.2)	<0.05
How shall we help if an infant is severely choking and is not able to breathe or cry?	315 (58.9)	218 (34.6)	<0.05
What will be the chest compression to breath ratio while resuscitating an infant or newborn if there are 2 rescuers?	232 (43.4)	61 (9.7)	<0.05
Total Score (mean + SD)	6.3 (2.9)	3.5 (2.4)	<0.05

### Factors influencing CPR performance

Table 4 presents findings from both univariate and multivariate regression analyses on factors influencing individuals’ ability to perform CPR when confronted with the situation. Across various demographic variables, significant associations were observed. Males exhibited significantly higher odds of performing CPR compared to females, with univariate and multivariate analyses showing odds ratios (ORs) of 1.946 (95% CI 1.092–3.470) and 1.991 (95% CI 1.071–3.701), respectively. Participants from medical faculties also had higher odds of performing CPR compared to those from non-medical faculties, though the effect was more pronounced in univariate analysis (OR 3.001, 95% CI 1.623–5.550 vs. 1.000) compared to multivariate analysis (OR 1.633, 95% CI 0.774–3.443). Notably, individuals who had received CPR training demonstrated significantly increased odds of performing CPR in both univariate (OR 3.826, 95% CI 2.117–6.714) and multivariate (OR 2.745, 95% CI 1.315–5.732) analyses compared to those without training.

**Table 4:** Factors influencing ability to perform CPR when confronted with the situation

Variables		Ability to perform CPR when confronted with the situation			
		Univariate regression		Multivariate regression	
		OR 95% (C.I)	*P* value	OR 95% (C.I)	*P* value
Age	Below or equal to 20	1 (r)	0.143	1 (r)	0.725
	Above 20	1.546 (0.863–2.767)		1.187 (0.457–3.088)	
Sex	Female	1 (r)	<0.05	1 (r)	<0.05
	Male	1.946 (1.092–3.470)		1.991 (1.071–3.701)	
Faculty	Non-Medical	1 (r)	<0.001	1 (r)	0.198
	Medical	3.001 (1.623–5.550)		1.633 (0.774–3.443)	
Marital status	Single	1 (r)	0.631	1 (r)	0.441
	Engaged	1.553 (0.527–4.576)		1.103 (0.323–3.773)	
	Married	1.553 (0.348–6.940		2.879 (0.569–14.571)	
Academic year	First year	1 (r)	<0.05	1 (r)	0.433
	Second year	1.632 (0.441–6.036)		1.259 (0.327–4.840)	
	Third year	1.749 (0.505–6.057)		1.212 (0.327–4.490)	
	Fourth year	0.754 (0.122–4.664)		0.461 (0.059–3.586)	
	Fifth year	2.556 (0.611–10.689)		1.728 (0.317–9.410)	
	Sixth year	9.200 (1.237–68.408)		5.270 (0.525–52.874)	
	Intern	5.520 (1.283–23.756)		2.591 (0.455–14.741)	
Have you ever received a course or training on CPR?	No	1 (r)	<0.001	1 (r)	<0.05
	Yes	3.826 (2.117–6.914)		2.745 (1.315–5.732)	

### Confidence levels and sufficiency perception

**Figure 1.**
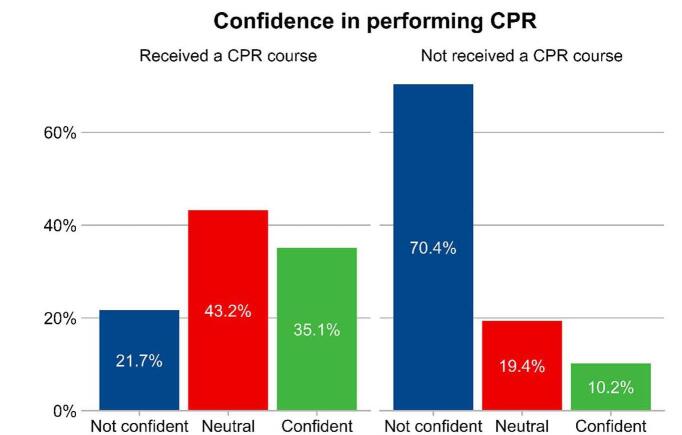
Confidence in performing CPR among students who received a previous training and who did not receive.

**Figure 2.**
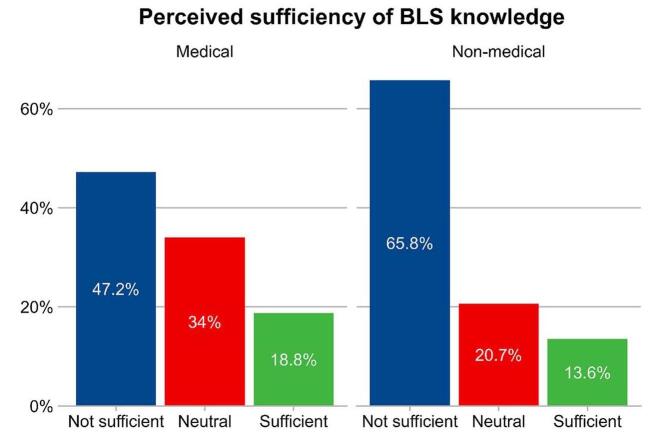
Perceived sufficiency of basic life support knowledge among medical versus non-medical students.

Figure 1 illustrates the stark contrast in confidence levels for performing CPR between students who received prior training and those who did not. Among students with previous CPR training, over a third (35.1%) felt confident in their ability to administer CPR, while only 10.2% of those without training shared this level of confidence. Conversely, the majority (70.4%) of students without CPR training reported feeling “not confident,” compared to just 21.7% of those who had received the training. Figure 2 highlights the differences in the perceived sufficiency of BLS knowledge between medical and non-medical students. Medical students generally have a more positive perception of their BLS knowledge compared to non-medical students. A lower percentage of medical students (47.2%) felt their BLS knowledge was not sufficient, compared to a higher percentage of non-medical students (65.8%) who felt the same way. Conversely, a higher percentage of medical students (18.8%) felt that their BLS knowledge was sufficient, compared to 13.6% of non-medical students.

## Discussion

Our study assessed BLS awareness and knowledge among undergraduates at our university, revealing several key insights into the current state of CPR training and its impact on student confidence and competence in life-saving techniques. Our study found that a significant proportion of the participants (71.2%) believed that CPR training should be mandatory. Despite this belief, only 29.6% of participants had received CPR training. Participants from medical faculties had higher odds of performing CPR and greater BLS knowledge compared to their non-medical counterparts. Notably, participants who had received CPR training had significantly higher BLS knowledge scores and confidence in performing CPR.

Our findings revealed that females had significantly higher BLS scores compared to males (4.9 vs. 4.5, *P* < 0.05), which is consistent with studies conducted in Saudi Arabia^[22]^ and Egypt^[18]^, where females also demonstrated better knowledge and attitudes toward CPR. Our findings, however, contradict those of a single medical faculty study in Egypt^[19]^, where males had significantly higher BLS scores compared to females. These results suggest a potential gender disparity in BLS knowledge that warrants further investigation to understand the underlying factors contributing to these differences.

A substantial proportion (70%) of our participants believed that CPR training should be mandatory, yet only 29.6% reported having received such training. This gap between belief and actual training mirrors findings from two studies in Saudi Arabia^[21,22]^ and Jordan^[23]^ which reported a similar percentage of participants receiving BLS training. These results pale in comparison to those from countries that place high importance on BLS training, such as the United Kingdom^[24]^, where 80% of students reported completing a BLS course as part of their undergraduate medical studies. The significant difference in BLS knowledge scores between trained and untrained (7.5 vs. 3.6, *P* < 0.05) underscores the critical impact of formal CPR training. This aligns with studies in Egypt^[25,26]^, Saudi Arabia^[21]^, Uganda^[27]^, and China^[28]^, where trained students exhibited significantly higher CPR and BLS knowledge and confidence.

Our study found that third-year students formed the largest group (38.4%), and participants from medical faculties demonstrated higher odds of performing CPR and greater BLS knowledge compared to their non-medical counterparts (6.3 vs. 3.5, *P* < 0.05). This pattern aligns with findings from Upper Egypt^[26]^ and the United Kingdom^[24]^, where fourth-year medical students had the highest CPR knowledge. The progressive increase in BLS knowledge with academic year and medical background highlights the importance of early and consistent integration of BLS training into the medical curriculum.

Confidence in performing CPR was significantly higher among those who had received training. Our results indicated that 35.1% of trained students felt confident in their ability to administer CPR, compared to only 10.2% of untrained students. This finding is consistent with a previous study in China^[28]^, where untrained respondents were more likely to lack confidence (69.6%). The positive correlation between training and confidence suggests that increasing access to CPR training could substantially improve students’ willingness and ability to perform CPR in emergencies.

A notable proportion of participants perceived their BLS knowledge as “insufficient.” Specifically, 70.4% of untrained students felt “not confident” about their BLS knowledge, compared to 21.7% of trained students. This gap was even wider among non-medical students, with 65.8% feeling their knowledge was insufficient compared to 47.2% of medical students. These findings align with a study conducted in Saudi Arabia^[29]^, where a significant portion of students felt their BLS knowledge was insufficient. These perceptions highlight the need for comprehensive and accessible BLS training programs to be implemented across all faculties, not just medical ones.

Our study showed that participants who had received CPR training had significantly higher BLS knowledge scores. This finding is supported by other studies, such as those conducted in Saudi Arabia^[21,22]^, Egypt^[25]^, Uganda^[27]^, and a multinational study of nine Arabic countries^[17]^, which reported better knowledge among those who had attended BLS courses. Furthermore, the sustained impact of CPR training on knowledge retention suggests that regular refresher courses could be beneficial in maintaining high levels of BLS competence among students, as supported by other studies^[30,31]^.

The study’s implications are significant for public health education and university policies. The clear benefit of CPR training on knowledge and confidence suggests that universities should consider mandatory CPR training for all students. This could be particularly impactful in non-medical faculties, where students currently have lower BLS knowledge and confidence. Furthermore, addressing the gender disparity in BLS knowledge may require tailored interventions to encourage male students to participate in CPR training.

Integrating mandatory BLS training into national educational policies could significantly enhance public preparedness for medical emergencies. For instance, in England, CPR training is part of the Health Education curriculum for secondary school students and is recommended for students aged 12 and above^[32]^. This initiative has led to higher rates of bystander CPR and improved survival rates in cases of out-of-hospital cardiac arrests^[33]^. Adopting similar policies in Egypt, particularly in non-medical faculties, could bridge existing knowledge gaps and contribute to reducing mortality from cardiac events and other emergencies, especially in low-resource settings where timely access to professional medical care may be limited.

### Limitations and recommendations

This study has several limitations that should be considered when interpreting the results. Firstly, using convenience sampling may limit the generalizability of the findings to all university students. The participants who chose to respond to the survey might have had a prior interest in or knowledge of BLS, which could introduce selection bias. Future research should consider employing stratified random sampling to ensure a more representative sample. Secondly, the self-reported nature of the questionnaire could introduce response bias, as participants might overestimate or underestimate their knowledge and skills. Incorporating objective measures, such as practical assessments of CPR skills, in future studies would help validate self-reported findings. Thirdly, the study’s cross-sectional design prevents us from establishing causality between CPR training and BLS knowledge or confidence. Longitudinal studies are recommended to track changes over time and better determine causal relationships. Additionally, the study was conducted at a single university, which may limit the applicability of the findings to other educational institutions with different student demographics or curricular emphases. Expanding the study to include multiple institutions across diverse regions could enhance its generalizability. Finally, while the questionnaire was based on a previously validated instrument, the specific adaptations for our study population may still have introduced measurement errors or affected reliability. Future research should prioritize additional validation steps for any adapted instruments to ensure their robustness in new contexts.

## Conclusion

This study highlights significant gaps in BLS knowledge and training among university undergraduates, with notable disparities based on gender, faculty, and prior training. Females demonstrated higher BLS scores than males, and students from medical faculties exhibited greater knowledge and confidence in performing CPR compared to their non-medical peers. Despite a strong belief in the necessity of CPR training, a large proportion of students had not received formal training, contributing to lower BLS knowledge and confidence levels. While these findings provide valuable insights, the study’s limitations must be considered. The use of convenience sampling may restrict the generalizability of the results, and the reliance on self-reported data may introduce response bias. Additionally, the cross-sectional design precludes the establishment of causality between CPR training and outcomes. Nonetheless, the findings emphasize the urgent need for mandatory and accessible CPR training programs across all university faculties. Incorporating periodic refresher courses and practical skill assessments could further enhance the effectiveness of BLS education. By addressing the identified gaps and adopting evidence-based training strategies, universities can better equip students with essential life-saving skills, ultimately contributing to improved emergency response readiness and reduced mortality in low-resource settings.

## Data Availability

The datasets generated and analyzed during the current study are available from the corresponding author upon reasonable request.

## Research registration unique identifying number (UIN)

This study is a survey-based research project and does not require registration in clinical trial databases. However, it adheres to ethical guidelines and IRB approval requirements.
